# Effects of contrast administration on cardiac MRI volumetric, flow and pulse wave velocity quantification using manual and software-based analysis

**DOI:** 10.1259/bjr.20170717

**Published:** 2018-01-16

**Authors:** Amir Fathi, Jonathan R Weir-McCall, Allan D Struthers, Brian J Lipworth, Graeme Houston

**Affiliations:** 1 Division of Molecular and Clinical Medicine, Medical Research Institute, University of Dundee, Dundee, UK; 2 Scottish Centre for Respiratory Research, Medical Research Institute, University of Dundee, Dundee, UK

## Abstract

**Objective:**

The aim of the current study was to determine the effects of gadolinium contrast agent on right (RV) and left ventricular (LV) volumetric, aortic flow and pulse wave velocity (PWV) quantification using manual, semi-automatic and fully automatic analysis techniques.

**Methods:**

61 participants free from known cardiovascular disease were recruited. Cardiac MR was performed on a 3 T scanner. A balanced steady-state free precession stack was acquired of the ventricles with phase contrast imaging of the aorta performed pre- and post-administration of 10 ml 0.5 mmol ml^−1^ gadoterate meglumine. The images were analysed manually, and using a semi-automated and a fully automated technique.

**Results:**

54 completed the study. Gadolinium-based contrast administration significantly increase the signal-to-noise ratio (pre: 830 ± 398* vs * post: 1028 ± 540, *p* = 0.003) with no significant change in contrast-to-noise ratio (pre: 583 ± 302* vs * post: 559 ± 346, *p* = 0.54). On LV analysis, post-contrast analysis yielded significantly higher end systolic volume (54 ± 20* vs *57 ± 18 ml, *p* = 0.04), and lower ejection fraction (59 ± 9* vs *57 ± 8%, *p* = 0.023). On RV analysis, gadolinium contrast resulted in no significant differences. Similar results were seen using the semi-automated and fully-automated techniques but with a larger magnitude of difference. Conversely, using both manual and software analysis aortic flow and PWV quantification proved robust to the effects of contrast agent producing only small non-significant differences.

**Conclusion:**

Gadolinium contrast administration significantly alters LV endocardial contour detection with this effect amplified when using semi-automated analysis techniques. In comparison, RV and PWV analysis is robust to these effects.

**Advances in knowledge:**

Contrast administration alters LV quantification but not flow analysis. However, these differences are small.

## Introduction

In recent years, cardiac MRI has played an increasingly integral role in the assessment of cardiovascular health and function with important implications for both diagnostic and quantitative evaluation purposes.^1–3^ Due to its high reproducibility, cardiac MR is considered the gold standard for the non-invasive quantification of both anatomical parameters such as myocardial mass, and functional parameters including ventricular volumes and aortic flow.^4–6^ Accurate quantification of these parameters is essential given the proven predictive capability for morbidity and mortality in patients suffering from coronary artery disease, cardiomyopathies and beyond.^7–9^ Similarly, the importance of MRI quantification of aortic stiffness using arch pulse wave velocity (PWV) has become increasingly prominent due to its ability to directly assess the central aortic stiffness rather than using peripheral substitutes.^10–12^

The use of gadolinium-based contrast agents in cardiac MR (CMR) has become a common place due to its use in the detection, characterization and quantification of myocardial scarring.^13–15^ Due to the need to wait 10 min after contrast administration to acquire late gadolinium enhancement sequences, acquisition of short axis cine images and aortic flow sequences are frequently obtained after the initial injection of gadolinium contrast to maximize the utilization of the scanner time and minimize downtime. However, without exception normal reference ranges for ventricular quantification have been published based on sequences obtained without contrast agent.^16^ A literature search on the effects of contrast agents yielded conflicting results with some studies showing no changes in ventricular parameters before and after contrast administration while others reported a significant impact up on left ventricular (LV) quantification.^17–19^ In light of the small cohorts and the lack of right ventricular (RV) analysis within said studies, the validity of comparing values obtained after the administration of gadolinium-based contrast agents with non-contrast sequences is potentially questionable.

The effects of contrast agents on PWV analysis are similarly unknown. Furthermore, there is no evaluation of the effects of contrast agents on contour detection software, which are commonly used for post-processing purposes. Thus, to better delineate the effects of contrast agents on cardiac MR-based volumetric and flow quantification, this study aims to: (i) assess the effects of contrast agent on both LV and RV stroke volume (SV), end-systolic volume (ESV), end-diastolic volume (EDV), ejection fraction (EF) and left ventricular mass (LVM); (ii) to determine the effects of contrast on aortic flow and PWV measurements; (iii) to compare the effects of gadolinium contrast on these measures obtained using manual and automated techniques.

## Methods and materials

### Study population

The study recruited 61 study participants (mean age: 66.1 ± 9.0, 51.9% male, body mass index 27.9 ± 5.2 kg m^–^^2^) free from clinically apparent cardiovascular disease from the general population, following advertisement of the project in a University distributed newsletter. All images were reviewed and screened for evidence of regional wall motion abnormality or remodelling by an experienced radiologist, as well as careful analysis of EF to ensure absence of silent ventricular systolic dysfunction. All participants provided written informed consent for the study, which was conducted in accordance with the Declaration of Helsinki and was approved by a research ethics committee.

### Cardiac MR acquisition

Cardiac MR images were obtained using a 3 T scanner (3T Magnetom Prisma Siemens Medical Solutions, Erlangen, Germany) with a 32-channel cardiac coil. Sequences were obtained for the quantification of ventricular mass and volumes and aortic flow both before and after contrast administration.

**Figure 1.**
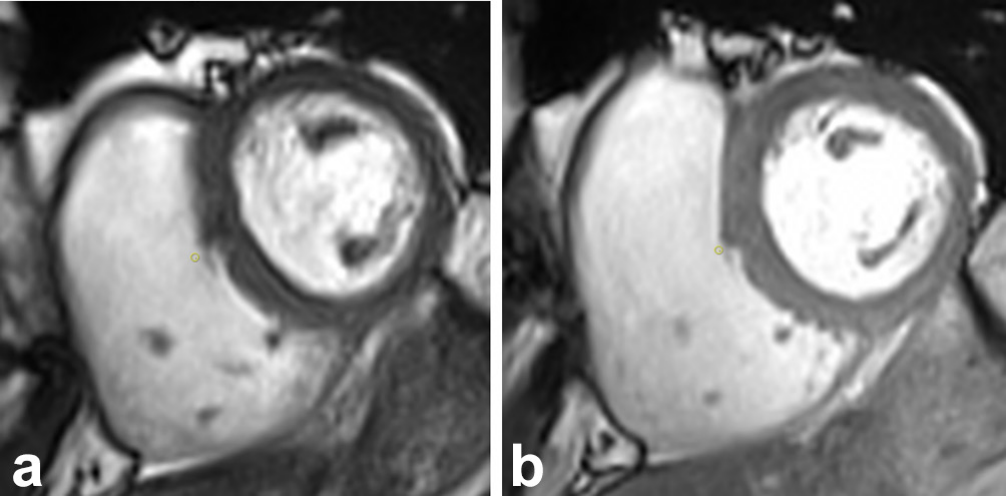
Mid ventricular end-diastolic short axis cine images acquired (a) pre- and (b) post-administration of contrast.

For the ventricular acquisition, a short axis stack was planned aligned to the atrioventricular groove. A balanced steady-state free precession acquisition was then performed in breath-hold from the atrioventricular ring to the apex (Figure 1). Slice thickness = 6 mm, interslice gap = 4 mm, time to recovery/time to echo = 47.6/1.49 ms, no. averages = 1, Phases = 25, bandwidth per pixel = 446 Hz, flip angle = 53°, field of view (FOV) = 360 × 360 mm^2^, FOV Phase = 84.4%, matrix = 256 × 256, parallel acceleration factor = 2, slice per breath-hold = 1.

**Figure 2.**
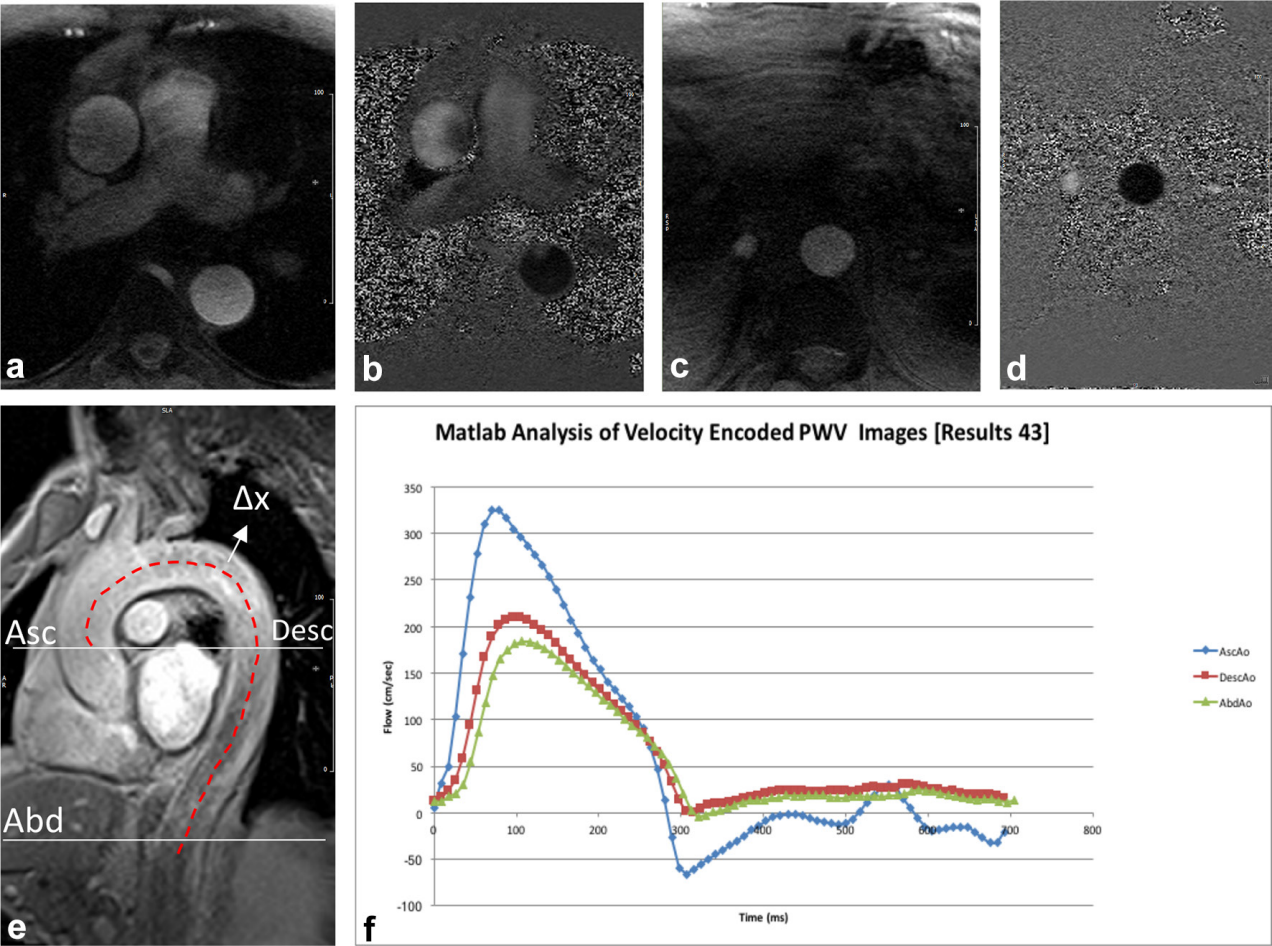
Aortic flow and PWV calculation: magnitude and phase images through the aortic arch are demonstrated in panels (a, b) respectively from which the ascending and descending aortic flow curves are obtained (f). Abdominal aorta magnitude (c) and phase (d) images were used to calculate the abdominal waveform. Panel (e) illustrates method used to derive ∆x between the three sites, while Panel (f) demonstrates the flow waveforms at these sites used to calculate ∆t. PWV, pulse wave velocity.

**Figure 3.**
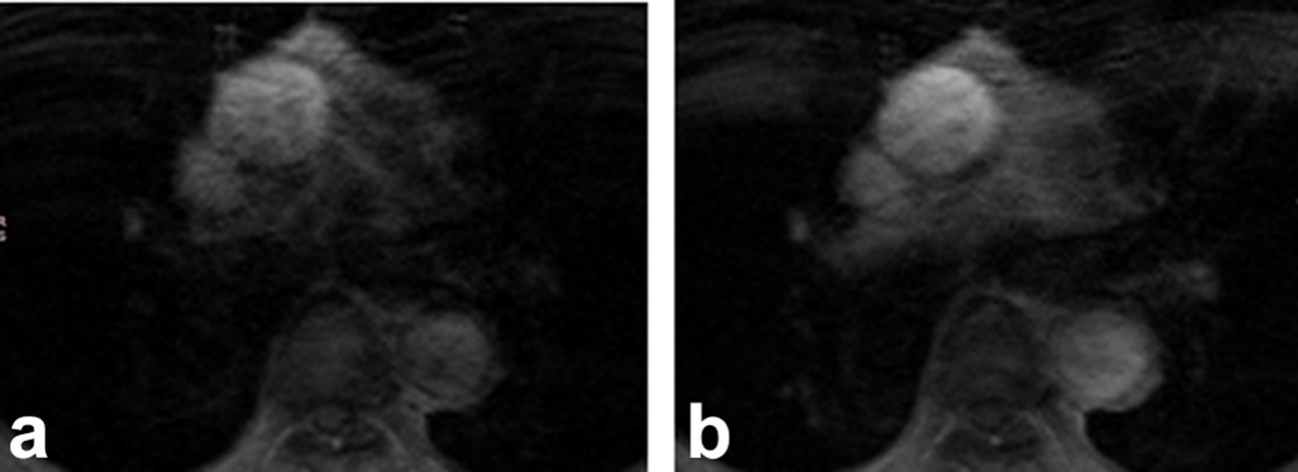
Representation of ascending aorta for calculating flow and PWV; slices at the level of right pulmonary artery acquired (a) pre- and (b) post-administration of contrast. PWV, pulse wave velocity.

For aortic flow acquisition, a “candy-cane” view of the aorta was first acquired to allow both slice planning and subsequent interslice distance measurement. Two phase-contrast sequences were planned, the first at the level of the right pulmonary artery to be perpendicular to the ascending aorta, the second just below the level of the diaphragm (Figure 2). The phase-contrast sequence (Figure 3) was then acquired in free-breathing with the following acquisition parameters: slice thickness = 6 mm, time to recovery/time to echo = 12/4 ms, no. averages = 1, Phases = 80, velocity encoding = 150 cm s^–1^, bandwidth per pixel = 340 Hz, flip angle = 15°, FOV = 320 × 320 mm^2^, matrix = 512 × 512.

After the acquisition of these sequences was completed, 10 ml 0.5 mmol ml^−1^ gadoteric acid (Dotarem, Guerbet, France) was administered at 1.5 ml s^−1^ through a cannula in the antecubital fossa followed by a 20 ml saline flush administered at 1.5 ml s^−1^. After 2 min (to avoid the early significant changes in arterial enhancement post-contrast administration) the ventricular (Figure 1) and aortic sequences (Figure 3) were repeated using the same alignment and acquisition parameters. The cine stack was acquired over approximately 5–7 min depending on the number of slices required, while the phase contrast sequence took approximately 4 min depending on heart rate.

### Ventricular analysis

Ventricular analysis was performed as per Society of Cardiovascular Magnetic Resonance guidelines.^20^ All analysis was performed using a commercially available software package Circle CVI42 (Calgary, Canada).

#### Three analytical methods for ventricular quantification

**Figure 4.**
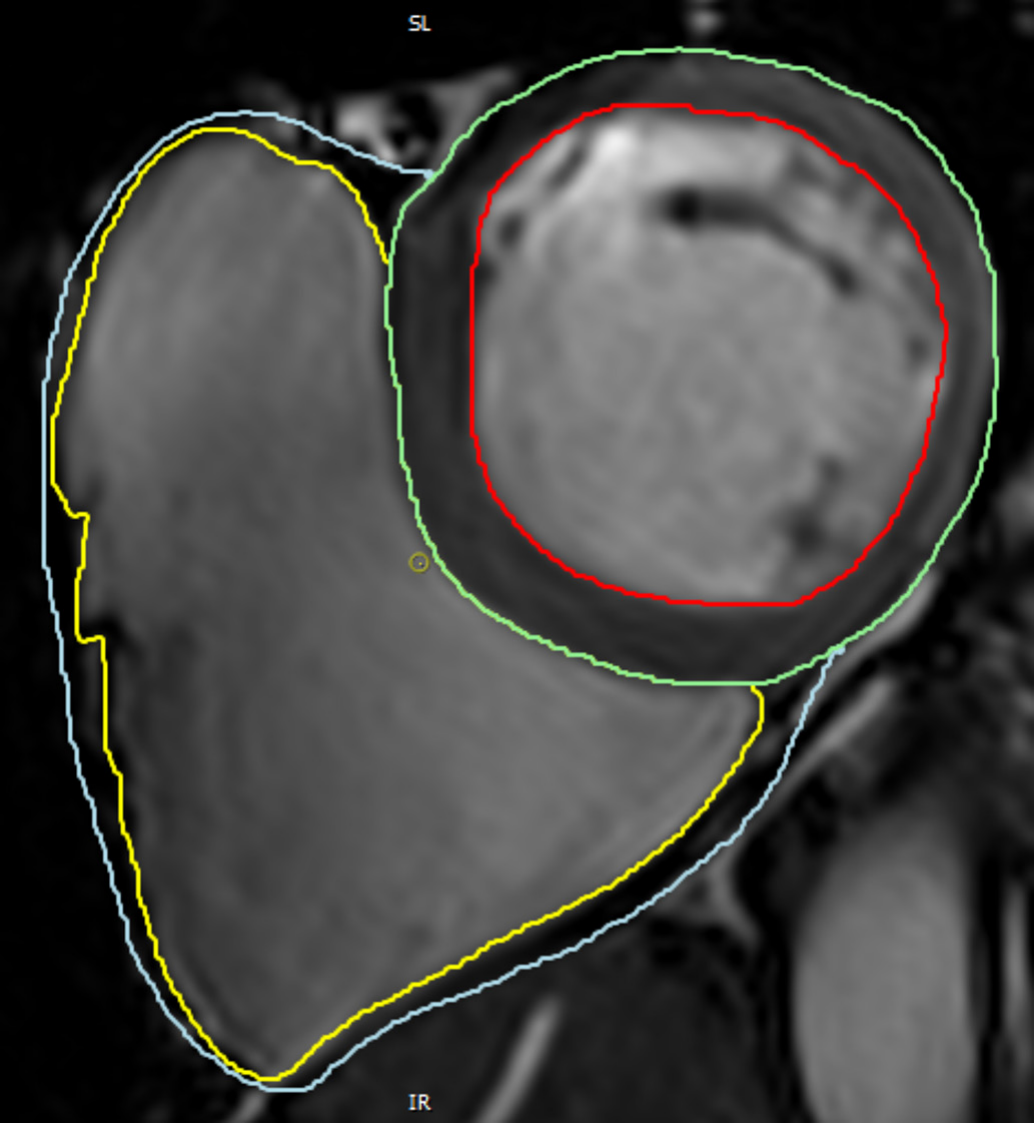
Right and left ventricular endocardial and epicardial contours.

Manual analysis (MA), semi-automated analysis (SA) and “fully” automated analysis (FA). For the purpose of MA, epicardial and endocardial contours were drawn around the right and left ventricles at end systole and end diastole by a radiologist with 5 years of cardiac MRI experience. The septum was treated as belonging to the left ventricle for LV mass quantification. Papillary muscles and trabeculations were included in the LV cavity volume and excluded from the LV mass quantification. SA was achieved by manually positioning the cursor over a central region within the ventricle in a single slice at end-diastole, thereafter, the signal intensity thresholding tool was used to grow an endocardial contour towards the edge until the entirety of the ventricular cavity was encompassed. Subsequently, the software generated an automatic epicardial contour. This process was repeated for each sequential slice. Manual adjustments were made where gross discrepancies were evident. For the FA, the mitral plane and ventricular apex were defined, after which the ventricular cavity was centred in the middle of the image and the automatic segmentation tool in circle used to automatically detect and draw the endocardial and epicardial contours. No subsequent adjustment was made to any contours generated using this technique. From the contours generated using these three techniques (Figure 4), the LV mass (LVM), LV and RV end-diastolic (EDV) and ESVs, LV and RV EFs and SV were calculated using the Simpson’s stack of discs technique.^21^

### Cardiac MR aortic flow acquisition

**Figure 5.**
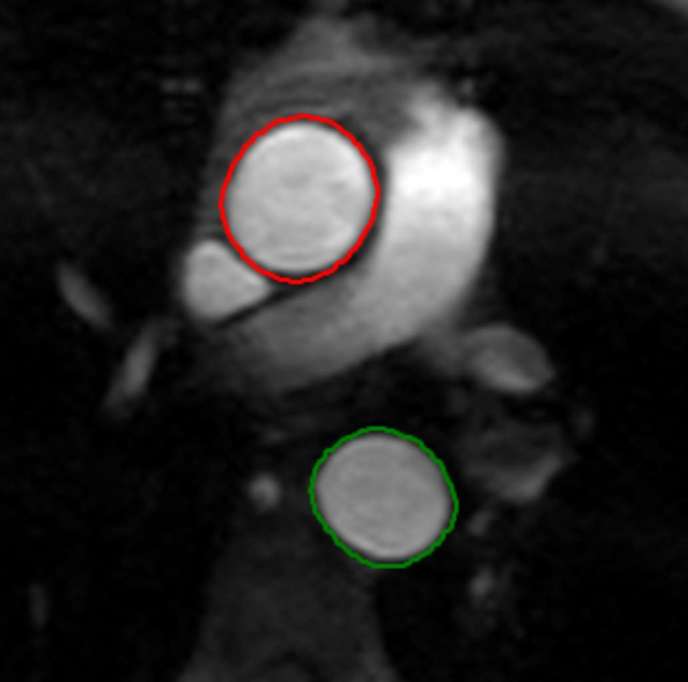
Representation of contours within the ascending and descending aorta.

Aortic flow was calculated using a manual (MA) and semi-automatic (SA) technique. For MA, a contour was drawn around the aorta and propagated throughout the cardiac cycle. Each image was then manually adjusted to ensure accurate contouring of the aorta. For SA analysis, a signal intensity thresholding tool was used to grow a region of interest (ROI) to encompass the lumen of the vessel then automatically propagated throughout the remainder of the cardiac cycle. Only images where the contour included either the superior or inferior vena cava, pulmonary artery or other major vessel were adjusted. This process was repeated for the ascending, descending and abdominal aorta. The ascending aorta contour was used for aortic flow calculation (Figure 5). For PWV calculation, the transit time technique was used as previously described, using the formula^22^

$$PWV=\frac{d}{\mathrm{§\#x0394;}t}$$

where “*d*” is the difference in distance between the sites of flow measurement and “Δ*t*” is the difference between the arrival time of the flow waves at the two sites. This was determined using the intersection of the baseline flow with a line drawn through the data points in the 20–80% portion of the systolic upstroke.^23^ To calculate *d*, the sagittal-oblique candy-cane view of the aorta was used to measure (i) the distance between the ascending and descending aorta and (ii) distance from the descending to abdominal aorta. Arch PWV was calculated as: the time delay between flow waves arriving at the ascending and descending thoracic aorta (Δ*t*), and the distance between these two points on the candy-cane view (d). To compute the aortic PWV, the time delay between the arrival of the flow wave within the ascending thoracic aorta and the arrival within the abdominal aorta was used in combination with the distance between the two (calculated by summing the distance between the plane through the ascending and descending aorta, and the distance from the descending aorta to the abdominal aorta plane).

### SNR and CNR measurements^24^

**Figure 6.**
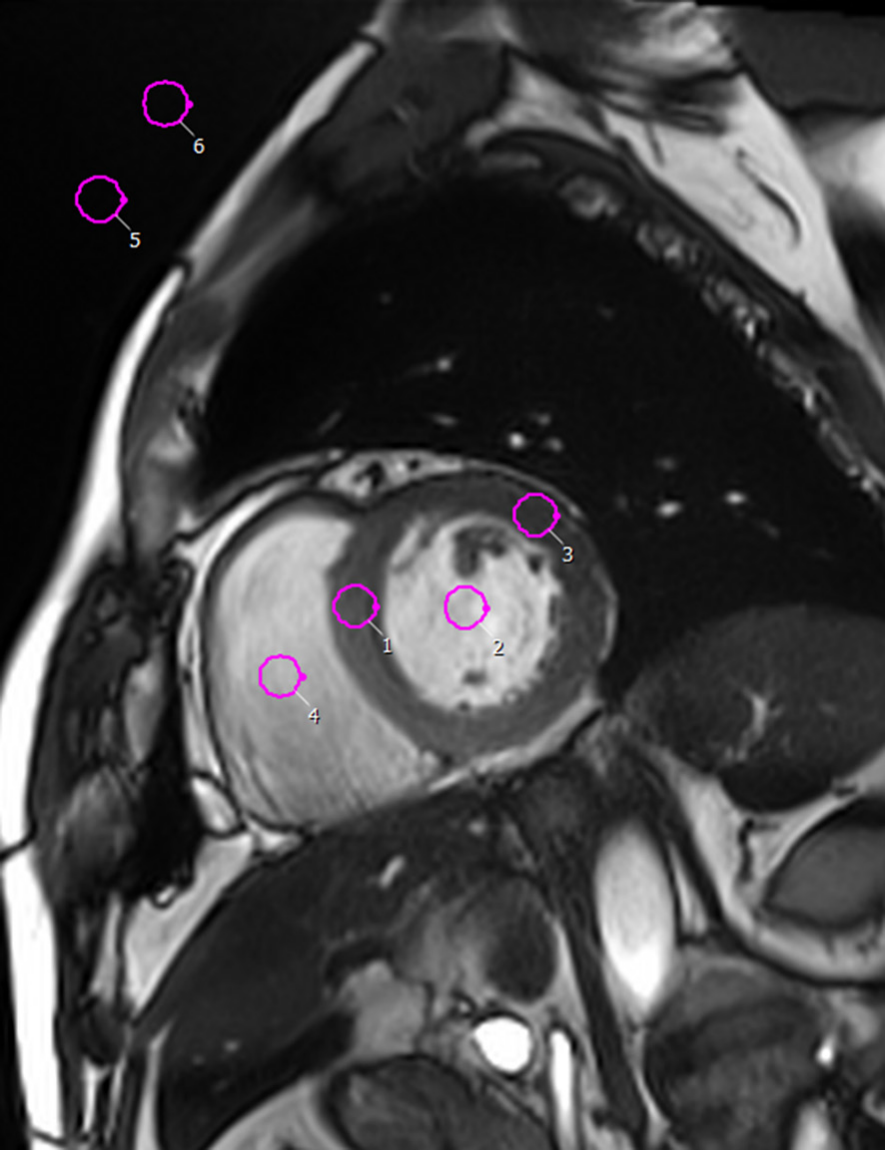
Illustration of method for SNR and CNR measurements; ROIs  are indicated by circles drawn within the ventricular cavity (2 and 4), the myocardium (1 and 3) and outside the image (5–6). CNR, contrast-to-noise ratio; ROI, region of interest; SNR, signal-to-noise ratio.

For ventricular signal-to-noise ratio (SNR) and contrast-to-noise ratio (CNR) quantification, four ROIs were drawn within the heart (RV cavity, LV cavity, septal wall and lateral wall) and two outside of the image area (Figure 6). All SNR and CNR measurements were taken at end-diastole. SNR was calculated by dividing the average signal from the two ROI within the ventricular cavities by the standard deviation of ROI outside the heart as follows: [(RV blood pool mean + LV blood pool mean)/2] / [(Air 1 SD + Air 2 SD)/2]. CNR was calculated as follows: {[(RV blood pool mean + LV blood pool mean)/2] – [(lateral wall mean + septal wall mean)/2]}/air SD.^25, 26^ For the phase contrast SNR, two ROIs were drawn in the ascending and descending aorta on the first magnitude image of the stack. Two further ROIs were drawn outside the body. SNR was calculated as: [(ascending aorta + descending aorta)/2] / [(Air 1 SD +Air 2 SD)/2].

### Repeatability and reproducibility

Repeated measurements were made by the same experienced observer in 20 participants following a time interval of greater than 3 months between the first and second reading. These 20 were selected randomly from those free from cardiac or respiratory motion artefact on all sequences so as to ensure that any differences in repeatability were due to the effects of contrast rather than variances in the image quality.^27^ Quantitative differences between measurements 1 (original) and 2 (repeat analysis) were analysed using Bland–Altman plots.

### Statistical analysis

Continuous data were presented as mean ± SD, while nominal data was reported as N (%). Normality of distribution and equality of variances were tested. Comparison of pre- and post-gadolinium contrast measurements was performed using a paired-sample *t*-test. Correlation of continuous variables was performed using a Pearson’s correlation. Bland–Altman plots were generated to assess repeatability of the analysis pre- and post-contrast administration. A *p* < 0.05 was considered statistically significant for differences between the measures. All statistical analysis was performed using SPSS (IBM SPSS Statistics for Windows, v. 22.0, NY).

## Results

From the original 61 participants, 54 completed the imaging protocol, with 7 participants lost due to claustrophobia or discomfort precluding any scanning, or abandonment of the scan acquisition prior to the administration of contrast agent due to technical issues.

### Left ventricular analysis

Administration of gadolinium contrast agent led to an increased SNR (pre: 830 ± 398 *vs* post: 1028 ± 540, *p* = 0.003) with no significant change in the CNR (pre: 583 ± 302 *vs* post: 559 ± 346, *p* = 0.54) calculations.

The results from MA found LVESV was significantly higher (54 ± 20* vs *57 ± 18 ml, *p* = 0.039) and LVEF significantly lower (59 ± 9 * vs * 57 ± 8%, *p* = 0.023) following the administration of gadolinium contrast agent (Table 1 for comparison of ventricular volumes pre- and post-administration of contrast). Similar results were observed in SA, showing a post gadolinium-contrast increase in LVESV (48 ± 20* vs *53 ± 21 ml, *p* = 0.000) and a reduction in LVEF (62 ± 10 * vs * 60 ± 9%, *p* < 0.001). In comparison, the FA, gadolinium contrast produced statistically significant differences with a higher LVEDV (117.2 ± 32* vs *127.41 ± 31; *p* < 0.001), and LVESV (62.4 ± 21* vs *69.5 ± 26; *p* < 0.001), with a lower LVM (110.6 ± 23* vs *105.4 ± 25; *p* = 0.030).

**Table 1.** LV ventricular analysis: manual, semi-automated and fully-automated analysis of LV parameters comparing measurements before and after administration of contrast agent

**Technique of analysis**	**Pre-contrast**	**Post-contrast**	***p* (<0.05)**
Manual			
LVEDV (ml)	130.8 ± 31.0	131.7 ± 29.8	0.54
LVESV (ml)	54.1 ± 20.1	57.0 ± 17.9	0.04
LVSV (ml)	76.6 ± 19.6	74.8 ± 18.8	0.17
LVEF (%)	59.0 ± 9.5	57.0 ± 8.1	0.02
LVM (g)	105.4 ± 28.6	106.7 ± 25.6	0.38
Semi-automated			
LVEDV (ml)	126.4 ± 31.4*^a^*	129.2 ± 31.0*^a^*	0.06
LVESV (ml)	48.3 ± 19.9*^a^*	52.6 ± 20.8*^a^*	<0.001
LVSV (ml)	78.0 ± 19.7	76.6 ± 18.3	0.15
LVEF (%)	62.1 ± 9.6*^a^*	59.8 ± 9.5*^a^*	<0.001
LVM (g)	109.1 ± 27.3*^a^*	108.6 ± 26.2	0.69
Fully automated			
LVEDV (ml)	117.2 ± 32.13^*a*^	127.41 ± 31.4*^a^*	<0.001
LVESV (ml)	62.4 ± 21.8*^a^*	69.5 ± 26.1*^a^*	<0.001
LVSV (ml)	54.8 ± 18.8*^a^*	57.9 ± 17.6*^a^*	0.21
LVEF (%)	46.9 ± 10.9*^a^*	45.9 ± 12.4*^a^*	0.49
LVM (g)	110.6 ± 23.8*^a^*	105.4 ± 25.2	0.03

*^a^*Significant differences found in software-based volumetric analysis when compared with manual analysis (Supplementary Material 1, Supplementary material available online). Student’s *t*-test with significance level of <0.05. Pre- and post-contrast refers to the absence and presence of contrast agent respectively. LV, left ventricular; LVEDV, left ventricular end-diastolic volume; LVESV, left ventricular end-systolic volume; LVSV, left ventricular stroke volume; LVEF, left ventricular ejection fraction; LVM, left ventricular mass.

Administration of contrast agent produced no significant difference in manual RV quantification (Table 2). Conversely, RVEDV (124.7 ± 35.6* vs *136.6 ± 38.7, *p* ≤ 0.001) and RVESV (53.8 ± 21.6* vs *62.9 ± 25.4, *p* ≤ 0.001) were noted to increase significantly in SA post contrast administration analysis, whereas RVEF was significantly reduced (57.4 ± 8.7* vs *54.8 ± 8.4, *p* = 0.01). Similarly, FA analysis with contrast agent showed a rise in RVEDV (140.8 ± 42.3* vs *153.4 ± 50.7, *p* = 0.01) and RVESV (68.3 ± 32.7* vs *90.9 ± 36.4, *p* ≤ 0.001) and a decrease in RVEF (51.8 ± 14.4* vs *40.5 ± 14.7, *p* ≤ 0.001).

**Table 2.** RV ventricular analysis: manual, semi-automated and fully automated analysis of RV parameters comparing measurements before and after administration of contrast agent

**Technique of analysis**	**Pre-contrast**	**Post-contrast**	***p* (<0.05)**
Manual			
RVEDV (ml)	129.4 ± 35.3	130.5 ± 35.2	0.66
RVESV (ml)	54.3 ± 22.8	54.6 ± 23.3	0.87
RVSV (ml)	75.2 ± 19.6	75.7 ± 18.7	0.74
RVEF (%)	58.6 ± 8.5	59.3 ± 10.4	0.60
RVM (g)	36.9 ± 8.9	35.1 ± 10.8	0.13
Semi-automated			
RVEDV (ml)	124.7 ± 35.6*^a^*	136.6 ± 38.7^*a*^	<0.001
RVESV (ml)	53.8 ± 21.6	62.9 ± 25.4^*a*^	<0.001
RVSV (ml)	70.9 ± 19.6*^a^*	73.7 ± 19.3	0.14
RVEF (%)	57.4 ± 8.7	54.8 ± 8.4^*a*^	0.01
Fully automated			
RVEDV (ml)	140.8 ± 42.3*^a^*	153.4 ± 50.7^*a*^	0.01
RVESV (ml)	68.3 ± 32.7^*a*^	90.9 ± 36.4^*a*^	<0.001
RVSV (ml)	72.5 ± 29.4	62.6 ± 32.9^*a*^	0.06
RVEF (%)	51.8 ± 14.4^*a*^	40.5 ± 14.7^*a*^	<0.001

^*a*^Significant differences found in software-based volumetric analysis when compared with manual analysis (Supplementary material). Student's *t*-test with significance level of <0.05. Pre- and post-contrast refer to the non-contrast images and images acquired following contrast administration respectively. RV, right ventricular; RVEDV, right ventricular end-diastolic volume; RVESV, right ventricular end-systolic volume; RVSV, right ventricular stroke volume; RVEF, right ventricular ejection fraction; RVM, right ventricular mass.

### Right * vs * left ventricular stroke volume quantification

In absence of contrast agent, SA found a significant difference between RVSV and LVSV (mean difference = 7.12 ± 10.57 ml, *p* < 0.001), which disappeared post-administration of gadolinium contrast dye (mean difference = 2.87 ± 10.23 ml, *p* = 0.66). These findings were also seen on FA analysis (pre-contrast mean difference = −17.71 ± 24.64, *p* < 0.001; post-contrast mean difference = −4.71 ± 30.30, *p* = 0.30). In comparison, RVSV and LVSV were in agreement (Table 3) when measured manually both before (mean difference = 1.42 ± 7.0, *p* = 0.18) and following contrast administration (mean difference = −0.94 ± 5.61, *p* = 0.27). SA analysis showed significant correlation between RVSV and LVSV both pre-contrast (*r* = 0.86, *p* ≤ 0.001) and post administration of contrast agent (*r* = 0.85, *p* < 0.001). FA analysis showed a moderate correlation between RVSV and LVSV both pre (*r* = 0.55, *p* < 0.001) and post (*r* = 0.41, *p* = 0.005) contrast administration. No significant differences were observed on MA (pre *r* = 0.94, *p* < 0.001; post *r* = 0.96, *p* < 0.001).

**Table 3.** Evaluation of the effects of contrast agent and technique used (manual, semi-automated and fully-automated) in quantification of right and left ventricular stroke volume

Technique of analysis	LVSV	RVSV	*p* (<0.05)	*^a^r* (*p*-value)
Manual				
Pre-contrast	76.6 ± 19.6	75.2 ± 19.6	0.18	0.94 (<0.001)
Post-contrast	74.8 ± 18.8	75.8 ± 18.8	0.27	0.96 (<0.001)
Semi-automated				
Pre-contrast	78.1 ± 19.7	71.0 ± 19.6	<0.001	0.86 (<0.001)
Post-contrast	76.6 ± 18.3	73.7 ± 19.3	0.07	0.85 (<0.001)
Fully automated				
Pre-contrast	54.8 ± 18.7	72.5 ± 29.4	<0.001	0.55 (<0.001)
Post-contrast	57.9 ± 17.6	62.6 ± 32.9	0.30	0.41 (0.005)

*^a^r* = two-tailed Pearson’s correlation. LVSV, left ventricular stroke volume; RVSV, right ventricular stroke volume.

### Aortic flow quantification

Gadolinium contrast resulted in a statistically significant increase in aortic SNR (pre: 36.6 ± 18.7 *vs* post: 44.2 ± 22.3, *p* = 0.001).

Gadolinium contrast administration produced a non-significant difference in MA of aortic flow (pre-contrast: 62.3 ± 12.8; post-contrast: 60.7 ± 12.3, *p* = 0.06), with no difference observed in semi-automatic analysis (*p* = 0.14). There was no significant change in aortic PWV using either manual or semi-automatic techniques (*p* > 0.05 for all; Table 4). However, semi-automatic technique yielded consistently lower whole aorta PWV than MA (manual = 8.1 ± 2.1 m s^–1^; semi-automatic = 7.5 ± 1.9 m s^–1^, *p* < 0.001) with similar observation seen post-contrast administration (manual = 8.9 ± 4.2 m s^–1^; semi-automatic = 7.7 ± 1.8 m s^–1^, *p* = 0.03).

**Table 4.** Analysis of the effects of contrast agent and technique used (manual *vs* semi-automated) in quantification of aortic flow and PWV

**Technique of analysis**	**Pre-contrast**	**Post-contrast**	***p* (<0.05)**
Manual			
Aortic flow (ml)	62.3 ± 12.8	60.7 ± 12.3	0.06
Arch PWV (ms^−1^)	7.9 ± 2.0	8.2 ± 2.6	0.61
Aortic PWV (ms^−1^)	8.1 ± 2.1	8.9 ± 4.2	0.20
Semi-automated			
Aortic flow (ml)	59.6 ± 12.1*^a^*	58.2 ± 11.8*^a^*	0.14
Arch PWV (ms^−1^)	7.3 ± 1.8*^a^*	7.7 ± 2.2	0.21
Aortic PWV (ms^−1^)	7.5 ± 1.9*^a^*	7.7 ± 1.8*^a^*	0.45

^*a*^Denotes semi-automatic quantified parameters which were significantly different (*p* ≤ 0.05) in comparison with manual analysis. PWV, pulse wave velocity.

In post-gadolinium contrast administration, there were no significant changes in heart rate (pre: –73.6 ± 22.4 * vs *post: 72.7 ± 20.0 bpm, *p* = 0.67), systolic blood pressure (pre: –129 ± 20 * vs * post: 129 ± 18 mmHg, *p* = 0.84) or diastolic blood pressure (pre: –74 ± 8 * vs *post 76 ± 9, *p* = 0.06).

### Reproducibility

**Figure 7.**
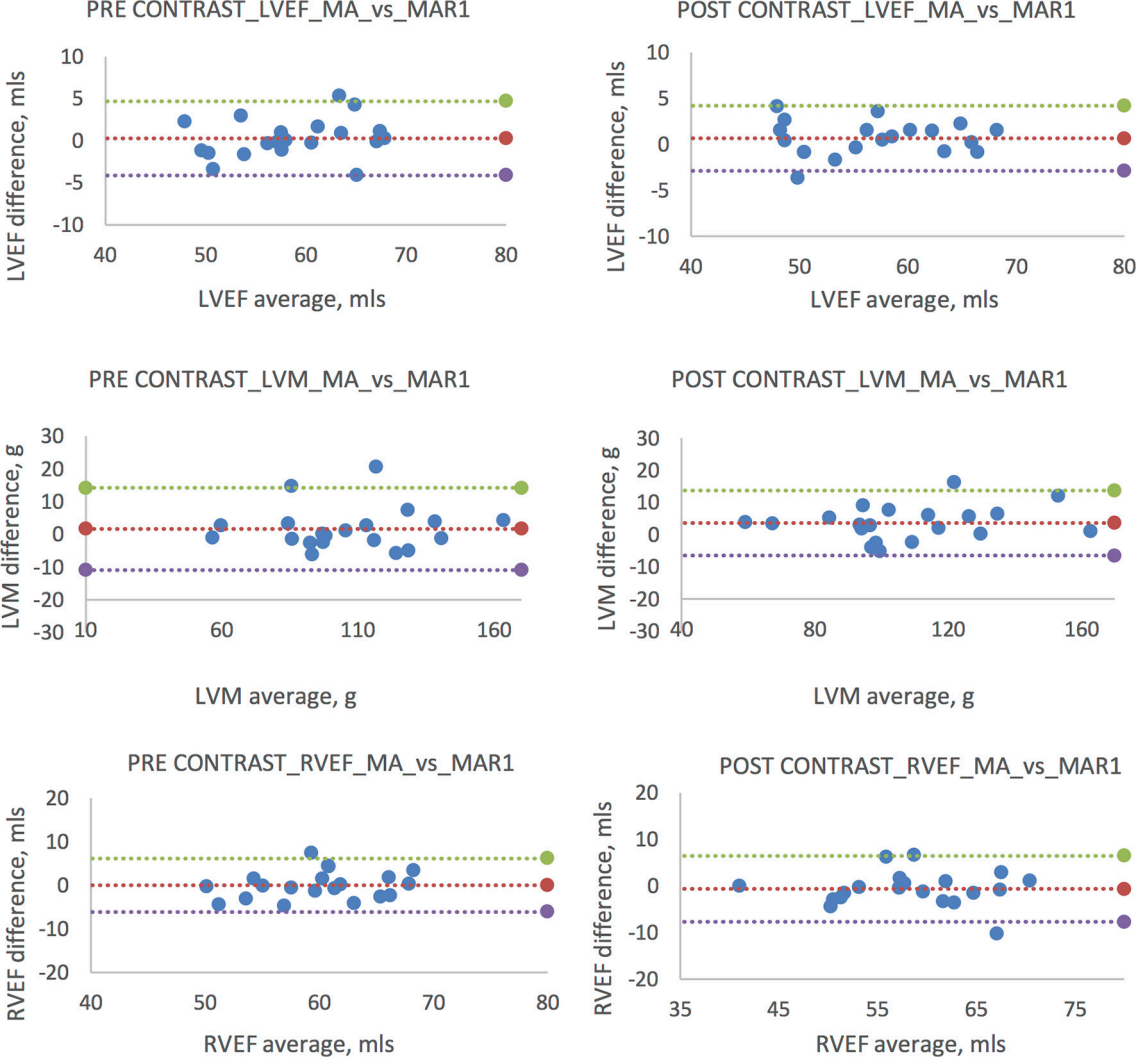
Bland–Altman plots comparing manual pre- * vs * post-contrast intraobserver reproducibility for left and right ventricles. Individual plots comparing original MA with repeat analysis (MAR1). Pre-contrast plots (left column) are paralleled with post-contrast (right column) counterpart. *In all subsequent Bland–Altman plots mean difference is represented by the middle line with the upper and lower lines representing a ± 2 SD limit. LVEF, left ventricular ejection fraction; LVM, left ventricular mass; MA, manual analysis; SD, standard deviation.

**Figure 8.**
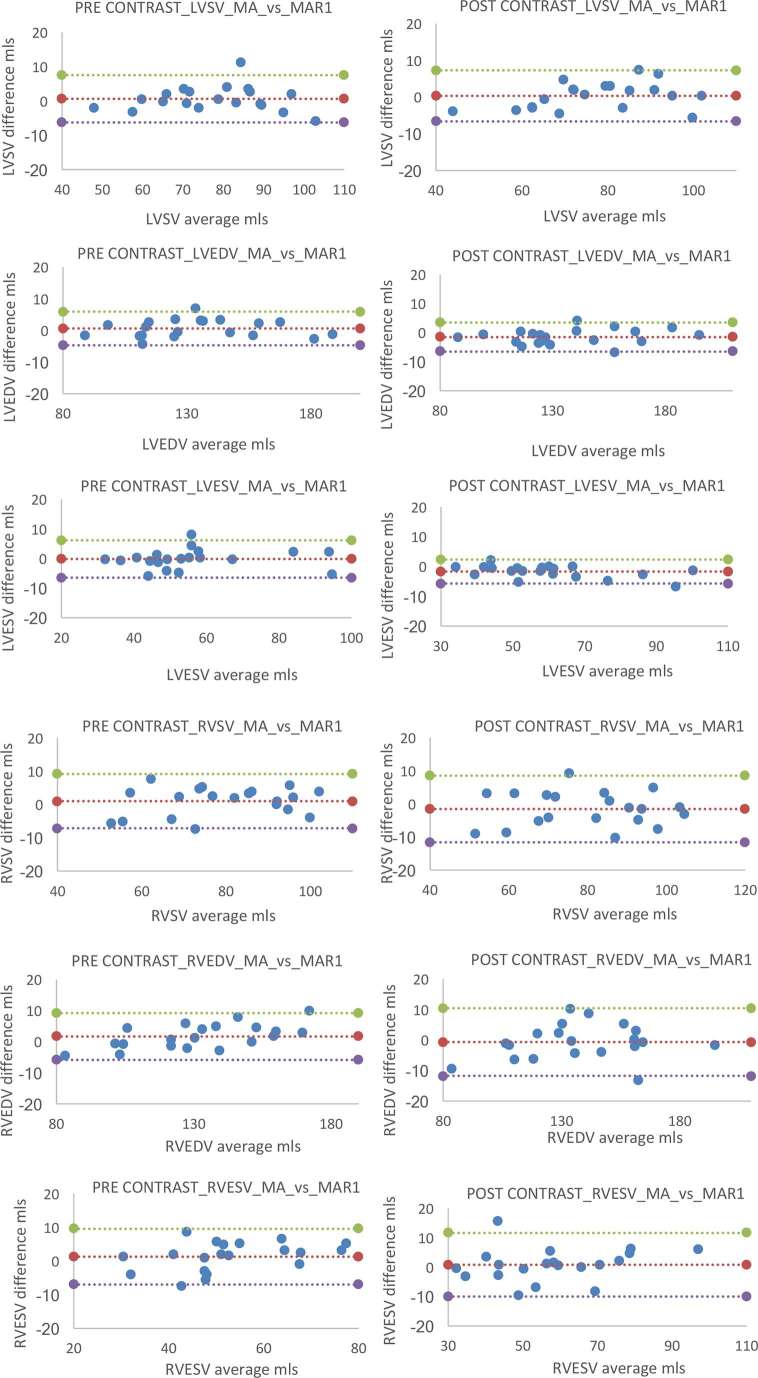
Bland–Altman plots comparing manual pre- * vs * post-contrast intraobserver reproducibility for left and right ventricular SV, EDV and ESV. Individual plots comparing original MA with repeat analysis (MAR1). Pre-contrast plots (left column) are paralleled with post-contrast (right column) counterpart. EDV, end-diastolic volume; ESV, end-systolic volume; LVEDV, left ventricular end-diastolic volume; LVESV, left ventricular end-systolic volume; LVSV, left ventricular stroke volume; MA, manual analysis; RVEDV, right ventricular end-diastolic volume; RVESV, right ventricular end-systolic volume; RVSV, right ventricular stroke volume; SV, systolic volume.

**Figure 9.**
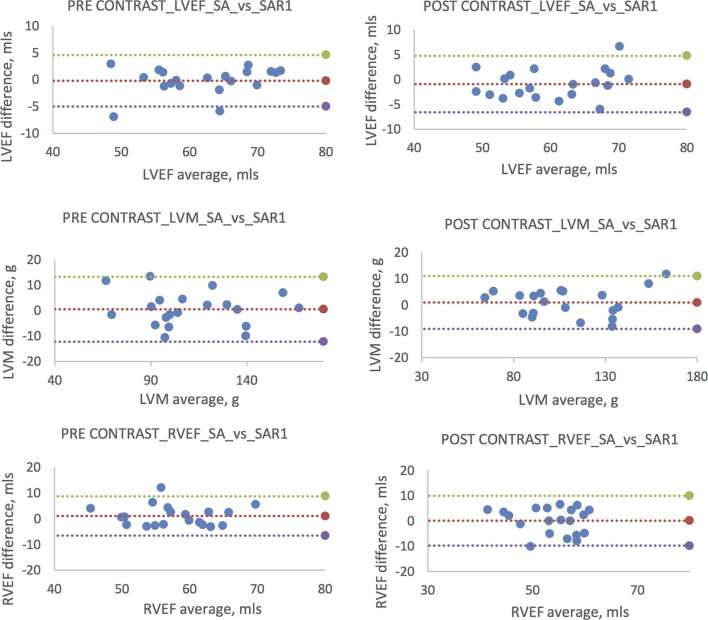
Bland–Altman plots comparing semi-automated pre- * vs * post-contrast intraobserver reproducibility for left and right ventricles. Individual plots comparing original SA with repeat analysis (SAR1). Pre-contrast plots (left column) are paralleled with post-contrast (right column) counterpart. LVEF, left ventricular ejection fraction; LVM, left ventricular mass; RVEF, right ventricular ejection fraction; SA, semi-automated analysis.

**Figure 10.**
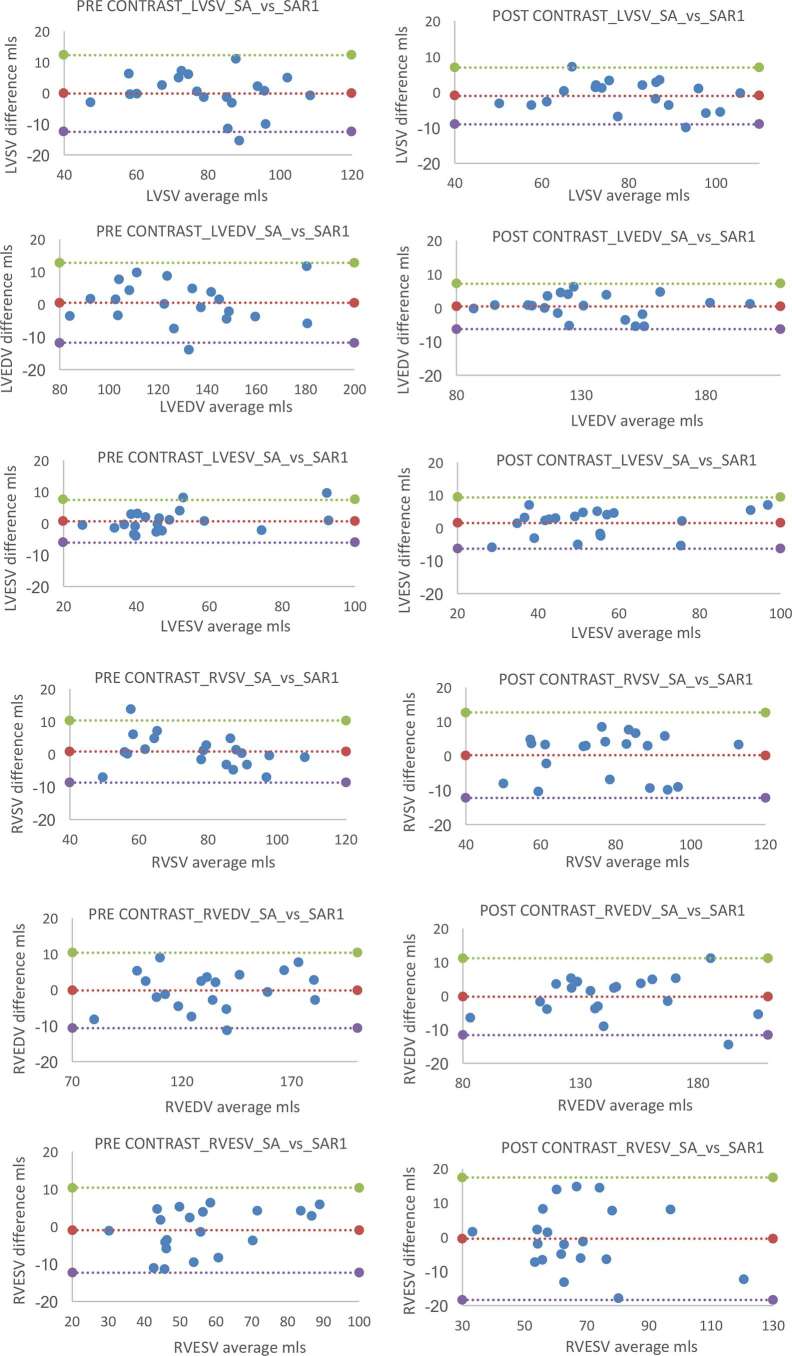
Bland–Altman plots comparing manual pre- * vs * post-contrast intraobserver reproducibility for left and right ventricular SV, EDV and ESV. Individual plots comparing original manual analysis (SA) with repeat analysis (SAR1). Pre-contrast plots (left column) are paralleled with post-contrast (right column) counterpart. EDV, end-diastolic volume; ESV, end-systolic volume; LVEDV, left ventricular end-diastolic volume; LVESV, left ventricular end-systolic volume; LVSV, left ventricular stroke volume; RVEDV, right ventricular end-diastolic volume; RVESV, right ventricular end-systolic volume; RVSV, right ventricular stroke volume; SV, systolic volume.

**Figure 11.**
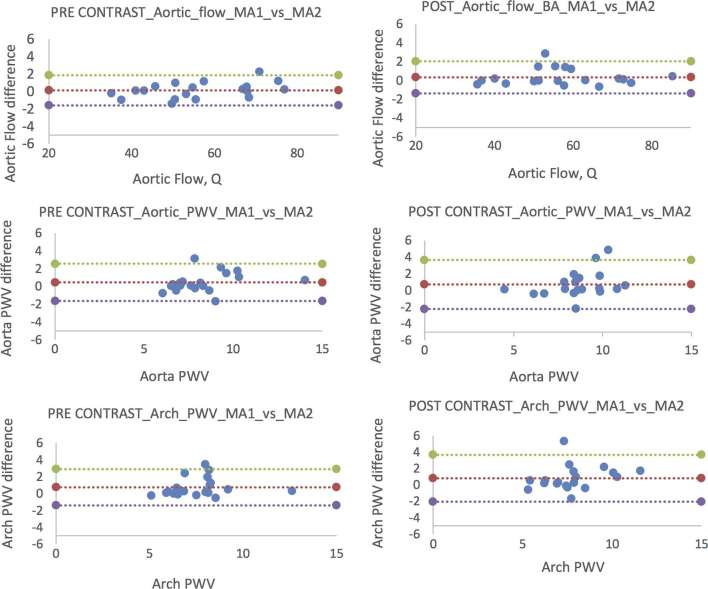
Bland–Altman plots comparing manual pre * vs * post-contrast intraobserver reproducibility for aortic flow and PWV. Shown here are plots comparing original manual data (MA1) with repeat analysis (MA2); total aortic flow (a) aortic PWV (b) and aortic arch PWV (c). Pre-contrast plots (left column) are paralleled with post contrast (right column) counterpart. PWV, pulse wave velocity.

**Figure 12.**
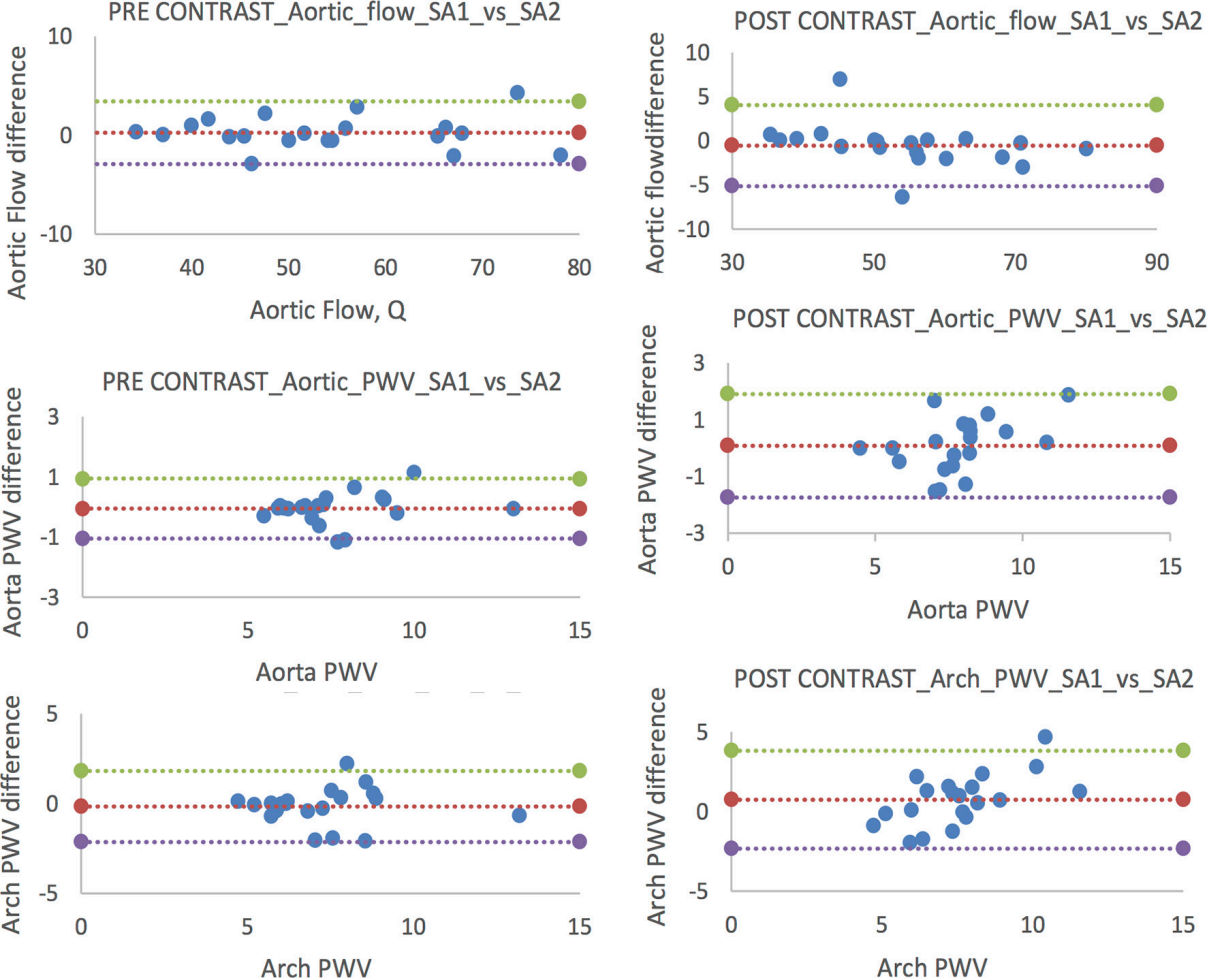
Bland–Altman plots comparing semi-automated pre- * vs * post-contrast intraobserver reproducibility for aortic flow and PWV. Shown here are plots comparing original semi-automated data (SA1) with repeat analysis (SA2); total aortic flow (a), aortic PWV (b) and aortic arch PWV (c). Pre-contrast plots (left column) are paralleled with post-contrast (right column) counterpart. PWV, pulse wave velocity.

Analysis of images post-contrast administration resulted in marginally wider limits of agreement for the intraobserver reproducibility of LVEF and RVEF, although there remained no significant difference between the readings (*p* > 0.05 for all). Figures 7–10 for Bland–Altman plots of left and right ventricular volumes, mass and EF. Aortic flow and PWV showed narrower limits of agreement indicating better repeatability on the pre-contrast sequences (Figures 11 and 12).

## Discussion

In the current study, we have found that contrast administration significantly improves SNR in both ventricular volumetric and aortic flow studies. Ventricular volumetric studies found no change in CNR. Increased post-contrast ventricular SNR resulted in significantly altered quantification of ventricular volumes and function, with these effects more pronounced using SA and FA techniques.

Our finding of an increase in LVESV with a resultant fall in LVEF is consistent with previous findings by Matthew et al, who also reported higher LVESV values following the delivery of contrast agent.^19^ Matthew et al also reported a decrease in LV mass following contrast administration, which we did not observe in the current study.^19^ Similar to our own, this previous study used a 3 T magnet, and the image acquisition parameters were broadly the same, thus these are unlikely to account for the observed differences in the studies. However, Matthew et al included papillary muscle and trabeculations where they were in direct contact with the myocardium which we took care to exclude from LV myocardial mass. Thus, it may be that visualization of these small structures is impacted in a greater fashion than the larger more regularly shaped ventricular wall by the presence of contrast, this is supported by the greatest differences consistently being observed in ESV where differentiation of papillary muscles and trabeculations from the adjacent myocardium becomes more challenging. Advances in shimming technology in the past 5 years are particularly important at 3 T where flow and inhomogeneity-related artefacts are more problematic.^28^ Thus, the benefits of the improved SNR generated by contrast administration may become less pronounced as the underlying noise is gradually reduced by advancing imaging techniques and may account for the loss of some of the pre and post contrast differences between the two studies.

Conversely, Caspar et al purported no differences between pre- and post-contrast analysis.^17^ These incongruous findings may be due in part to differences in timing from contrast administration to commencement of imaging sequencing with the previous study acquiring their images immediately following the injection of contrast, which would include the early phases where there is significant alterations in contrast between the arterial, venous and myocardial compartments, while in the current study, we waited 2 min to achieve a more steady signal state within the myocardial chambers and myocardium. Different results may also have resulted from differing techniques in myocardial and blood pool segmentation, however, the method for the quantification of the mass and volumes in this previous study were not reported, thus comparison on this front is not possible. Finally, these differences may alternately be attributed to a discordance in the pharmacokinetics between the contrast agent used in the study by Caspar et al (gadobutrol) and the current study (gadoteric acid) with the former having a much shorter plasma half-life.^29^

Elsewhere, Krombach et al found contrast agent did not significantly alter LV volumes, however this study used a 1.5 T scanner where contrast has less effect on tissue relaxation compared with 3 T.

In addition, the current study is larger and thus better powered to detect small between group differences, which may explain the apparently conflicting findings. Furthermore, this work included three analysis techniques, all of which produce similar post contrast outcomes, further validating our current results.

We observed no significant differences in MA of the right ventricular volumes or function. LV quantification has received more attention than its neighbouring RV, with the effects of contrast agent on RV quantification not previously examined.^19^ The finding of a lack of significant difference in before and after contrast administration studies is reassuring, and adds robustness to the comparison of studies regardless of contrast administration status. However, this robustness did not hold true for a semi-automated technique utilizing a signal intensity threshold algorithm, where significant differences were observed in RVEDV, RVESV and RVEF. The fact that differences in LV and RV SV reduced post-contrast administration and agreement with manual RV quantification improved, indicates that the increased SNR provided by the administration of the contrast agent improves the detection of the thin RV wall and the discrimination between cavity and trabeculae.

The analysis of cardiac MR images using a semi-automated contour detection protocol is attractive due to reduction in analysis time and the potential for improved inter- and intraobserver reproducibility.^30, 31^ However, this is not without its challenges and limitations with capacity for error both during semi-automated detection (software error) and during manual adjustments (observer error). Ideally, the combination of software detection and manual correction by an observer may offer a more reliable and less time-consuming approach.^31^ This notion is supported by Jaspers et al^32^, who not only reported high correlations between manual and semi-automatic ESV, EDV and EF, but also found that the semi-automatic technique provides careful distinction between muscular structures and ventricular blood pool.

In the current work, we only allowed adjustments for major contouring errors such as extension of the contours into adjacent non-ventricular structures by the software, but made no adjustments for minor errors such as inclusion/exclusion of trabeculae within the cavity. This is of course not the practice in the clinical realm where all contours will be closely scrutinized, however, was necessary to determine the true effects of contrast agent on software ventricular analysis. Allowing greater manual adjustment may have minimized the differences and thus, minimized the effects of contrast agent, however such adjustments would simply dilute the effects of the contrast on the software, and start to replicate the results of a MA, if every minor contour error were to be adjusted.

Multiple previous studies have demonstrated both an improved SNR, phase-to-noise ratio and velocity-to-noise ratio for phase-contrast imaging post-administration of contrast.^33–35^ Despite these findings, previous studies have found no significant difference in absolute flow quantification, a finding in contradistinction to our own finding of a slight increase in aortic flow post-contrast administration.^14^ The differences in aortic flow quantification, however, were small amounting to only a 2 ml (2.5%) difference before and after contrast administration. Conversely, SA analysis resulted in no such difference. This may reflect the difficulties in contouring the aorta during very early systole and when flow is slower leading to greater inaccuracy. It was noted by the authors that the software appeared to largely undersize the aorta during slower flow periods, which may make for a more consistent contour delineation allowing for measurements robust to small changes in contour visualization and detection and background noise. In comparison, PWV was unchanged post-contrast administration, and was not affected by the analysis technique used.

In our current study, we found a marked discrepancy between EF in manual *vs* fully automated quantification irrespective of the presence of contrast. Continuing advances in image processing and machine learning may advance this field in the future, but at present the current results suggest judicious review and contour correction is required following the automatic generation of endo and epicardial contours in the left and right ventricles.

There are limitations in the current study. There exists a time lag between images obtained without contrast and following contrast administration, thus the latter may be potentially prone to movement artefact due to developing participant discomfort. Additionally, the administration of 10 ml contrast agent and 20 ml saline could in theory change the measured volumes and flow due to change in volaemic status rather than the effects of the gadolinium on the generated RF signal. However, 30 ml is a small volume in healthy adults free from cardiovascular disease, and in fact, approximately matches the expected urinary output of 30–75 ml h^–^^1^. Secondly, even if this was the case, then the changes would still be real, just not by the expected mechanism. The dose of contrast agent administered was not weight-dependent in this study, which varies from routine clinical practice where a weight-based dosage is frequently used, however, we observed no significant relationship between CNR and weight, thus the confounding nature of this is likely limited. The current analysis was performed using a single post-processing software vendor, thus, further work is required to determine if these differences are observed using other software platforms. This is particularly pertinent with regards to the semi- and fully automated techniques, where proprietary software algorithms can differ substantially.

## Conclusion

Administration of contrast agent significantly alters endocardial contour detection regardless of technique used resulting in changes in ventricular quantification. This effect is amplified when using SA techniques, thus care must be taken when comparing values obtained before and after contrast administration and when comparing post-contrast volumes with reference values. PWV analysis remained robust to these differences.

## Availability of data and material

The datasets used and/or analysed during the current study are available from the corresponding author on reasonable request.
